# Respiratory *Klebsiella pneumoniae* infection transiently alters neutrophil responsiveness and enhances distal allergic inflammation

**DOI:** 10.3389/fimmu.2026.1853024

**Published:** 2026-08-04

**Authors:** Xilan Xiao, Jing Li, Tongqian Wu, Shirong Yan, Yan Wang, Rongtao Yang, Ziqian Guo, Wengui Gong, Zhicheng Hu, Fang Yu

**Affiliations:** 1 Center for Clinical Laboratories, Affiliated Hospital of Guizhou Medical University, Guiyang, China; 2 School for Laboratory Science, Guizhou Medical University, Guiyang, China; 3 Clinical Research Center, Affiliated Hospital of Guizhou Medical University, Guiyang, China

**Keywords:** allergic disease, bacterial infection, contact hypersensitivity, functional priming, neutrophil

## Abstract

**Background:**

Microbial exposure at respiratory interfaces shapes host defense, but whether prior localized bacterial infection can alter innate immune responsiveness and modulate subsequent allergic inflammation at distal sites remains unclear. This study investigated the impact of prior respiratory *Klebsiella pneumoniae* (KP) infection on distinct models of cutaneous allergic inflammation and the contribution of neutrophils to this cross-tissue modulation.

**Methods:**

Mice were intratracheally infected with KP subsequently subjected to passive cutaneous anaphylaxis (PCA), oxazolone-induced contact hypersensitivity, or fluorescein isothiocyanate (FITC)-induced contact hypersensitivity. Neutrophil depletion (anti-Ly6G) and adoptive transfer of bone marrow neutrophils from infected or naïve donors were performed. Neutrophil phenotype (CXCR2, CD62L) and function were assessed by flow cytometric analysis and ex vivo LPS-stimulated cytokine production (TNF-α, IL-1β, IL-6). Allergic responses were evaluated by ear swelling, vascular permeability (Evans blue), mast cell degranulation, histamine levels, and inflammatory cytokine profiles.

**Results:**

At day 7 after respiratory KP infection, bacterial loads in bronchoalveolar lavage fluid and blood were nearly undetectable, whereas inflammatory cytokines, including TNF-α, IL-1β, and IL-6, remained elevated in lung tissue, bronchoalveolar lavage fluid, and serum. Bone marrow neutrophils from KP-infected mice showed altered CXCR2 and CD62L expression and produced higher levels of TNF-α, IL-1β, and IL-6 after ex vivo LPS stimulation, suggesting enhanced neutrophil responsiveness during the post-infectious phase. Prior KP infection aggravated distal allergic inflammation in the PCA and oxazolone models, as shown by increased ear swelling, Evans blue extravasation, mast cell degranulation, histamine production, neutrophil infiltration, and inflammatory mediator expression. In contrast, the additional effect of KP infection was more limited in the FITC-induced contact hypersensitivity model, indicating a model-dependent response. In the PCA model, neutrophil depletion attenuated KP-enhanced allergic inflammation, whereas adoptive transfer of neutrophils from KP-infected donors restored and enhanced PCA responses more strongly than transfer of control neutrophils.

**Conclusion:**

Respiratory KP infection is associated with post-infectious neutrophil priming and enhanced distal allergic inflammation in a model-dependent manner. These findings suggest that prior bacterial respiratory infection may influence subsequent allergic responses through altered neutrophil responsiveness, with effects that are context-dependent rather than universal across all hypersensitivity models.

## 1 Introduction

Allergic diseases, characterized by aberrant IgE-mediated immune responses and excessive inflammation, have become a major global health burden (1, 2). Accumulating clinical and experimental evidence indicates that prior microbial exposure can profoundly modulate the susceptibility and severity of subsequent allergic diseases. One emerging mechanism underlying this effect is functional priming. Distinct from the long-term epigenetic reprogramming characteristic of trained immunity, functional priming represents a sustained, intermediate state of hyper-responsiveness wherein the residual inflammatory milieu following an initial pathogenic exposure induces a prolonged but reversible shift in the functional baseline of innate immune cells, thereby reshaping subsequent immune responsiveness during the post-infection recovery phase (3–6). Notably, such effects are not necessarily restricted to the site of the initial exposure. For example, skin exposure to *Staphylococcus aureus* can promote systemic sensitization to unrelated antigens (7, 8), and eosinophils have been shown to acquire innate immune memory following bacterial skin infection (9). However, the relationship between microbial exposure and allergic disease is complex and context-dependent: while certain commensal or environmental exposures promote tolerance (10, 11), severe pathogenic infections may instead prime the immune system for exaggerated responses (6, 12). The net outcome depends on factors such as the type, timing, and route of exposure, as well as the host’s inflammatory state (5, 13). However, how acute bacterial pneumonia influences distal allergic inflammation remains poorly understood.

*Klebsiella pneumoniae (KP)*, a common Gram-negative opportunistic pathogen, is a major cause of both community-acquired pneumonia and nosocomial infection (14). Beyond triggering acute pulmonary inflammation, KP infection can compromise the alveolar-capillary barrier, allowing bacterial components such as lipopolysaccharide (LPS) and locally produced inflammatory mediators to enter the circulation (15). In this way, respiratory KP infection may extend beyond the lung and reshape the systemic immune environment of the host (16, 17), potentially influencing inflammation in distant tissues.

Respiratory infections induce strong innate responses characterized by rapid recruitment and activation of neutrophils, which are essential for pathogen clearance but can also reshape systemic inflammatory states (18, 19). In this context, neutrophils represent a key but underappreciated link between localized infection and systemic immune modulation. As frontline and central innate effector cells in antibacterial defense, neutrophil development, maturation, and release are shaped by the surrounding pro-inflammatory milieu (20). Given the short lifespan, sustained functional alterations in neutrophils are unlikely to reflect intrinsic long-term memory, but may instead arise from transient post-infectious conditioning of myelopoiesis or systemic inflammatory signaling. Previous studies have already suggested that severe systemic inflammation can induce long-term alterations in myeloid cell function (21). Moreover, preliminary evidence has shown that gastrointestinal colonization of KP by oral gavage can aggravate local allergic symptoms in mice (22). We therefore hypothesize that respiratory KP infection might induce a transient post-infectious state characterized by functional priming of neutrophils, which in turn modulates the magnitude and quality of subsequent distal allergic responses. In this model, neutrophils act as central mediators linking pulmonary infection to peripheral inflammatory amplification.

The core pathological features of allergic disease include mast cell (MC) degranulation (23), neutrophil infiltration (24, 25), increased vascular permeability (26), and the release of mediators such as histamine and pro-inflammatory cytokines (27). Notably, bacterial component such as LPS can activate mast cells through toll-like receptors (TLRs), promoting the production of multiple inflammatory mediators, including tumor necrosis factor-α (TNF-α) and interleukin-13 (IL-13) (28, 29), and can further enhance IgE-mediated mast cell degranulation (30). These observations raise the possibility that prior KP infection may render immune cells more responsive to subsequent allergen challenge. In contrast to the tolerance-promoting effects proposed by the hygiene hypothesis (31), severe infection may instead impose a pro-inflammatory state that predisposes to exaggerated allergic responses.

To address this hypothesis, we established a respiratory KP infection model followed by multiple models of allergic inflammation, including passive cutaneous anaphylaxis (PCA), oxazolone-induced contact hypersensitivity, and FITC-induced contact hypersensitivity. These models were selected because they represent distinct allergic contexts, including IgE-mediated immediate hypersensitivity and hapten-induced delayed-type skin inflammation (32–36). These complementary systems allow us to assess whether prior respiratory infection alters allergic inflammation across distinct immunological contexts.

Importantly, while respiratory infection induces a complex inflammatory milieu involving multiple cytokines and effector cells, including mast cells and tumor necrosis factor-α (TNF-α) -associated pathways, our study focuses on defining the role of neutrophil functional alterations as a functionally and experimentally validated contributor to enhanced distal allergic inflammation.

## 2 Methods

### 2.1 Bacterial strains and growth condition

Bacterial strains used in this study were obtained from the Center for Clinical Laboratories, Affiliated Hospital of Guizhou Medical University. The primary strain was *K. pneumoniae* NTUH-K2044. For validation, two multidrug-resistant reference strains (ATCC BAA1705, designated KP1, and ATCC BAA2146, designated KP2) and *P. aeruginosa* PAO1 ATCC 15692 (non-*Klebsiella* control) were used. Strain characterization details are provided in the **Supplementary Materials**. Bacteria were incubated overnight in LB broth at 37 ˚C with 220 rpm, centrifuged at 13400 × g for 5 min, and then resuspended in fresh LB broth for growth until the mid-log phase (37).

### 2.2 Mice

All animal experiments were approved by the Ethics Committee of Guizhou Medical University. C57BL/6J mice were originally purchased from the Experimental Animal Center of Guizhou Medical University. Female C57BL/6J mice aged 6–10 weeks and weighing 20–25 g were used in all experiments. Mice were housed under specific pathogen-free conditions at 18 - 23°C and 40-60% relative humidity with a 12 h light/12 h dark cycle, with free access to food and water to ensure animal welfare. Mice were randomly assigned to experimental groups; and data collection and analysis were performed blinded to group allocation. All experiments were performed with at least three independent biological replicates, with the number of mice per group now explicitly stated in each figure legend.

### 2.3 KP-induced pulmonary infection model

Mice were anesthetized by intraperitoneal injection of Avertin (250 mg/kg) and intratracheally inoculated with 5 × 10^6^ CFU of *Klebsiella pneumoniae* in 50 µL phosphate-buffered saline (PBS). At 7 days post-infection (dpi), bronchoalveolar lavage was performed immediately after euthanasia. Euthanasia was performed by cervical dislocation under deep anesthesia to minimize suffering. Briefly, the trachea was exposed and cannulated with a blunt-ended needle, and the lungs were lavaged by repeatedly aspirating and instilling 0.2 mL of ice-cold sterile PBS. The recovered bronchoalveolar lavage fluid (BALF) was collected, kept on ice, and centrifuged at 300 × *g* for 5 min at 4°C. The supernatant was collected for bacterial load analysis immediately, and the remaining aliquots were stored at −80°C for subsequent cytokine measurement. Blood samples were collected, serum was collected and stored at −20°C. A portion of the lung tissue was snap-frozen in liquid nitrogen and stored for cytokine analysis.

All allergic challenge models were initiated at 7 days post-infection, a time point chosen to capture the post-acute phase after substantial bacterial clearance. At this stage, bacterial burdens in BALF and blood were nearly undetectable, whereas residual inflammatory cytokine elevation and lung histopathological changes remained detectable. This design allowed us to assess post-infectious immune modulation rather than the direct effect of ongoing bacterial infection.

### 2.4 Passive cutaneous anaphylaxis

Seven days post-infection, PCA was induced as follows (22), with slight modifications. Briefly, both ears of each mouse were intradermally injected with 25 µL of anti-DNP IgE (10 µg/mL; D8406, Sigma-Aldrich), and baseline ear thickness was measured. Twenty hours later, mice were intravenously injected 100 µL of DNP-HSA (1 mg/mL; A6661, Sigma-Aldrich) containing 2% Evans blue (Solarbio). After 30 min, the ears were photographed and ear thickness was measured again, after which mice were euthanized and tissues were collected. A full-thickness biopsy of the right ear was fixed for histological analysis, whereas the left ear was snap-frozen in liquid nitrogen and stored at −80°C for subsequent analysis. Blood was collected 12 h after antigen challenge as described above.

### 2.5 Neutrophil depletion and adoptive transfer

Neutrophil depletion was performed by intraperitoneal (i.p.) injection of 200 µg anti-Ly6G antibody (clone 1A8; BioXcell, BE0075-1) or an isotype control (rat IgG2a, clone 2A3; BioXcell, BE0089) in a 100-µL volume 24 h prior to sensitization. Neutrophil depletion efficiency was assessed in peripheral blood at the indicated experimental time point by flow cytometry. For adoptive transfer, 2 × 10^6^ neutrophils, isolated from the bone marrow of bacterially infected or uninfected donor mice, were resuspended in 200 µL of sterile PBS and intravenously (i.v.) injected via the tail vein 24 h post-depletion.

### 2.6 Contact hypersensitivity

CHS was induced as described previously (36), with slight modifications. On day 7 post-infection, 100 µL of 0.5% fluorescein isothiocyanate (FITC; Sigma-Aldrich, F7250) dissolved in a 1:1 acetone: dibutyl phthalate (Sigma-Aldrich, 524980) or 100 µL of 2% oxazolone (4-ethoxymethylene-2-phenyl-2-oxazolin-5-one, Ox; Sigma-Aldrich, 862207) in absolute ethanol was topically applied to the shaved abdominal region. On day 5 after sensitization, baseline ear thickness was measured with a micrometer followed by challenge with 15 µL of 0.5% FITC to both sides of one ear, 15 µL of 2% Ox to the dorsal side of the ear. Ear thickness was measured after 24 hours challenge, with baseline ear thickness being subtracted to obtain the change in ear thickness. Blood samples and ear tissues were collected as described above.

### 2.7 Evans blue extravasation

Evans blue was extracted from the remaining minced right ear tissue by incubation in formamide (D4551, Sigma-Aldrich) at 60°C for 24 h, and absorbance was measured at 610 nm.

### 2.8 Histology

Lung and ear tissues were collected and fixed overnight in 4% paraformaldehyde at room temperature, embedded in paraffin, sectioned at 5 μm, and deparaffinized before staining. Hematoxylin and eosin (H&E) staining was performed on lung and ear sections to assess tissue morphology and inflammatory infiltration. Toluidine blue staining was performed on ear sections to identify mast cells.

For immunofluorescence staining, ear sections were deparaffinized, rehydrated, and subjected to antigen retrieval in citrate buffer (pH 6.0) for 15 min. Sections were then permeabilized with 0.3% Triton X-100, blocked with 5% bovine serum albumin in PBS for 1 h at 37°C, and incubated with primary antibodies overnight at 4°C. After washing with PBS containing 0.1% Tween-20, sections were incubated with fluorochrome-conjugated secondary antibodies for 1 h at 37°C in the dark. Nuclei were counterstained with DAPI, and images were acquired using an Olympus fluorescence microscope.

Specifically, for mast cell degranulation analysis, toluidine blue stained ear sections were examined by two independent observers blinded to the experimental groups, with degranulated mast cells counted in at least five randomly selected high-power fields (400×) per section, and the percentage calculated as the number of degranulated cells divided by the total mast cells counted per field, with discrepancies resolved by consensus. Ear thickness measurements, histological scoring, and immunofluorescence analyses were also performed under blinded conditions.

### 2.9 Flow cytometry

The cell suspensions prepared from spleen and bone marrow were proceeded to flow cytometric analysis. 1 μl of prepared Zombie Aqua Fixable Viability Kit (BioLegend, Cat. No. 423101, USA) was added and incubated for 15 min at room temperature, 2 μL Fc receptors were blocked with an anti-mouse CD16/32 antibody for 20 min at 4°C. Subsequently, and then cells were incubated with 3 μL pre-titrated mixture of fluorochrome-conjugated antibodies [Anti-mouse CD45-PerCP (Biolegend; Cat#: 103129; Clone: 30-F11), Anti-mouse CD11b-PE/cy7 (Biolegend; Cat#: 101215; Clone: M1/70), Anti-mouse Ly-6G-FITC (Biolegend; Cat#: 127605; Clone: 1A8)] targeting specific surface markers for 30 min at 4°C in the dark. Following incubation, cells were washed twice with PBS/2% FBS, resuspended in an appropriate volume, and analyzed immediately on a flow cytometer (Navios, Beckman coulter) and data analyzed with FlowJo software (Tree Star Inc.).

### 2.10 Cytokine quantification

Excised ear tissues were weighed. A corresponding volume of ice-cold homogenization buffer (sterile PBS supplemented with protease inhibitors in 100:1) was added at a ratio of 10 μL per mg tissue. The tissue was finely minced on ice using sterile scissors and subsequently homogenized using a brief ultrasonication pulse on ice. The final homogenate was centrifuged at 12000 rpm for 10 min at 4°C to remove debris. The supernatant was aliquoted and stored at -80°C for subsequent analysis. Cytokines in the supernatants were quantified using a bead- based LEGENDplex immunoassay (Biolegend, 740446) according to the manufacturer’s protocol. Samples were analyzed on FACSCanto (BD, USA). Data were analyzed using LEGENDplex analysis software. For cytokine measurements, we confirm that concentrations in ear tissue homogenates were normalized to tissue weight, and expressed as pg per mg tissue (pg/mg). Ear tissues were weighed prior to homogenization, and this normalization strategy was consistently applied across all experimental groups to account for differences in tissue size and inflammatory edema.

### 2.11 Enzyme-linked immunosorbent assay

ELISA (Jianglai Biotechnology Co., Ltd., China) was performed according to the manufacture’s protocol to quantify the concentrations of TNF-α, IL-1β, and IL-6 in lung homogenates, bronchoalveolar lavage fluid (BALF), cell culture supernatants, and serum obtained from the infection model. Levels of histamine were determined in ear tissue homogenates, cell culture supernatants and serum from the allergy model using ELISA according to manufacturer’s instruction (BYabscience, China).

### 2.12 Isolation, identification and stimulation of neutrophils

From the femurs of infected and non-infected mice, the bone marrow cavities were flushed with PBS. Bone marrow neutrophils were isolated using a commercial kit (Solarbio, Beijing, China), and their purity was verified by flow cytometry for CD11b and Ly6G expression to be over 90% (**Supplementary Figure 1**), Cell viability was assessed by trypan blue exclusion and consistently exceeded 95%. Purified neutrophils were resuspended in RPMI 1640 medium supplemented with 10% fetal bovine serum. Equal numbers of viable neutrophils were seeded for each condition at 5 × 10^5^ cells per well and allowed to stabilize for 2 h before stimulation. Cells were then treated with 100 ng/mL lipopolysaccharide (LPS) or PBS for 4 h under identical culture conditions. Culture supernatants were collected, aliquoted, and stored at −20°C until cytokine measurement. Cytokine concentrations were measured in culture supernatants and compared between groups using the same initial number of viable neutrophils per well.

### 2.13 Statistical analysis

Data are presented as mean ± SEM unless otherwise stated. Biological replicates refer to individual mice, and the number of biological replicates is indicated in the figure legends. Technical replicates, when performed, were averaged before statistical analysis and were not treated as independent samples. Data normality was assessed using the Shapiro-Wilk test before parametric analysis. For comparisons between two groups, unpaired two-tailed Student’s t test was used for normally distributed data, whereas the Mann-Whitney U test was used for non-normally distributed data. For comparisons among more than two groups, one-way ANOVA followed by Tukey’s multiple-comparison test was used for normally distributed data; otherwise, the Kruskal-Wallis test followed by Dunn’s multiple-comparison test was used. No data were excluded unless predefined technical criteria were met, including sample loss, insufficient sample quality, failed model induction, or inadequate cell recovery for flow cytometric analysis. Statistical significance was defined as P < 0.05.

For cytokine analyses, each cytokine was analyzed independently. Statistical tests were selected based on the normality of each dataset, with non-parametric tests used when normality was not met. Cytokine grouping in the figures was for presentation only and did not indicate pooled analysis.

## 3 Results

### 3.1 *Klebsiella pneumoniae* infection amplified distal IgE-mediated allergic responses

**Figure 1:**
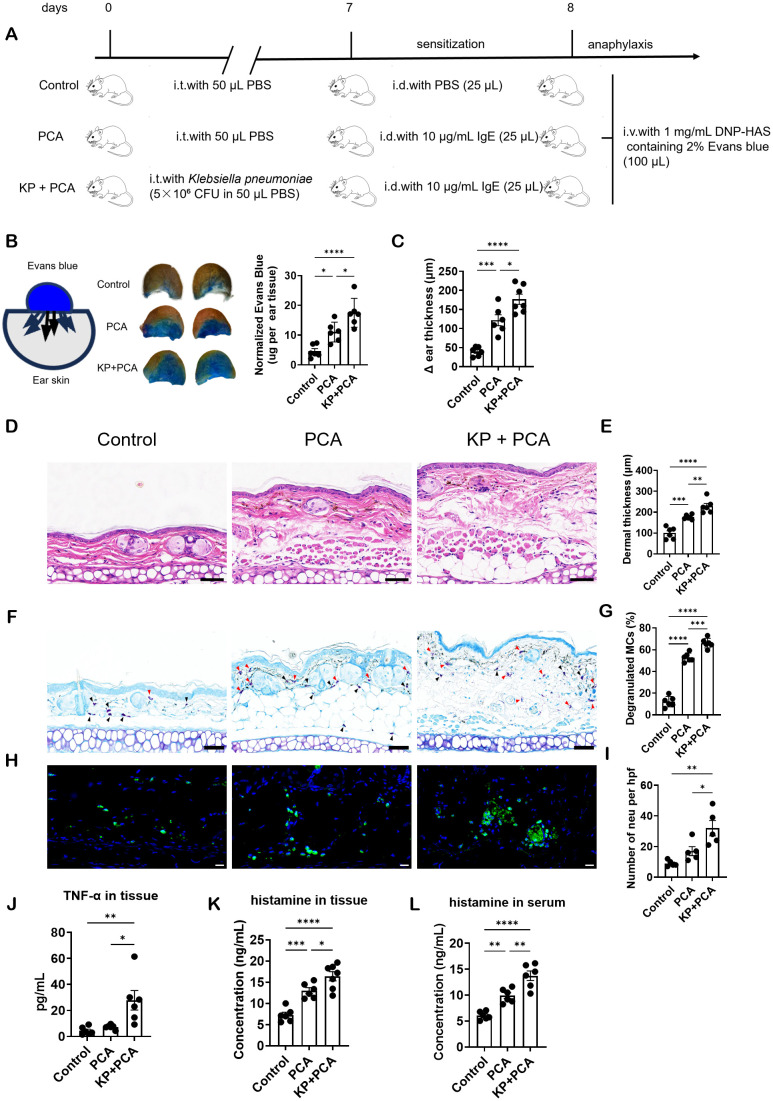
*Klebsiella pneumoniae* pretreatment augments IgE-elicited passive cutaneous anaphylaxis in the ear. **(A)** Schematic diagram of the experimental design. **(B)** Representative ear images and the quantification of Evans blue dye (n = 6/group). **(C–E)** Ear thickness **(C)** (n = 6 - 7), representative H&E- stained images of ears **(D)**, and dermal thickness **(E)** are shown (n = 6/group). Scale bars, 50 μm. **(F, G)** Representative Toluidine blue-stained images of MCs and the percentages of degranulated MCs in mouse ear (n = 6/group). Scale bars, 50 μm. **(H)** Immunofluorescent microscopic visualization with DAPI (blue) and Ly6G (green) to visualize neutrophils in ear skin. **(I)** Quantification of number of neutrophils in **(H)** is shown (n = 5/group). Scale bars, 50 μm. **(J, K)** Levels of TNF-α and histamine in mouse ear tissue [n = 6/group in **(J)**, n = 6–7 in **(K)**]. **(L)** Concentration of histamine in mouse serum (n = 6/group). The data are presented as mean ± SEM of three independent experiments. Control: PBS; PCA: PBS + IgE/DNP-HSA; KP + PCA: KP + IgE/DNP-HAS. PCA, passive cutaneous anaphylaxis; i.t., intratracheal; i.d., intradermal; i.v., intravenous; KP, *Klebsiella pneumoniae* (NTUH-K2044); MCs, mast cells; neu, neutrophil; TNF-α, tumor necrosis factor-α. Individual data points [**(B, C, E, G, I–L)**] represent data from a single animal. Significance was calculated using one-way ANOVA with Tukey’s multiple comparisons test [**(B, C, E, G)** and **(I–L)**]. *P < 0.05; **P < 0.01; ***P < 0.001; ****P < 0.0001.

To investigate whether respiratory infection influences peripheral allergic responses, we established a mouse model of IgE-mediated passive cutaneous anaphylaxis (PCA) following intratracheal *Klebsiella pneumoniae* (KP) infection (**Figure 1A**). Mice pre-exposed to KP exhibited significantly increased vascular permeability upon DNP-HSA challenge, as indicated by enhanced Evans blue dye extravasation compared to the PCA-only controls (**Figure 1B**). This was accompanied by increased ear swelling and dermal thickness (**Figures 1C–E**).

Histological analysis revealed a higher percentage of degranulated mast cells (MCs) in KP-pretreated mice (**Figures 1F, G**). In parallel, immunofluorescence staining showed increased neutrophil (Ly6G^+^) infiltration in the ear skin following challenge (**Figures 1H, I**). Consistently, KP-pretreated mice displayed elevated TNF-α levels in ear tissue and increased histamine concentrations in both tissues and plasma (**Figures 1J–L**), indicating an enhanced local inflammatory response following allergic challenge.

To further examine whether the observed phenotype was dependent on the bacterial strain or pathogen, we evaluated two additional *K. pneumoniae* isolates (KP1 and KP2, Detailed strain characterization information is provided in the **Supplementary Materials**) and included *Pseudomonas aeruginosa* as a non-*Klebsiella* respiratory pathogen. Because these experiments were performed as validation studies, only the PCA ear response readouts were assessed (**Supplementary Figure 3**). Similar to the original NTUH-K2044 strain, KP1 enhanced allergic responses in the PCA model. In contrast, KP2 showed a more limited phenotype, significantly increasing Evans blue extravasation without significantly affecting the other PCA readouts. Notably, prior respiratory infection with *P. aeruginosa* did not enhance allergic responses under the same experimental conditions. These findings suggest that the enhancement of distal allergic inflammation is not a universal consequence of respiratory bacterial infection and that both the magnitude and characteristics of the allergic response vary among different *K. pneumoniae* strains.

### 3.2 KP infection induces a transient post-infectious inflammation state associated with altered neutrophil phenotype and responsiveness

**Figure 2:**
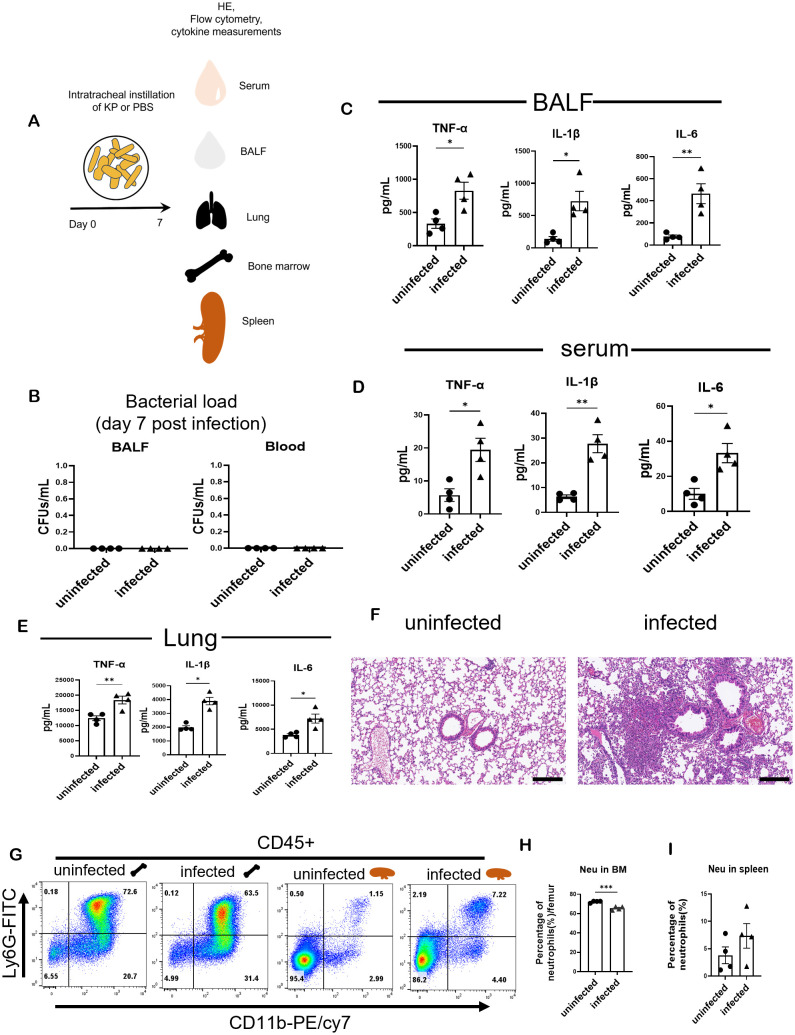
*Klebsiella pneumoniae* Infection Induces a Systemic Pro-Inflammatory Milieu. **(A)** Schematic diagram of the experimental design. **(B)** KP (NTUH-K2044) burden (CFUs) in BALF and blood on day 7 post infection (n = 4/group). **(C-E)** The level of inflammatory cytokines (TNF-α, IL-1β and IL-6) in BALF **(C)**, serum **(D)** and lung homogenate **(E)**, n = 4/group. **(F)** Representative H&E-stained images of lung 7 days after infection (n = 4/group). Scale bars, 200 μm. **(G–I)** Representative flow cytometric dot plots and the percentage of neutrophils in bone marrow and spleen (n = 4/group). BALF, bronchoalveolar Lavage Fluid; TNF-α, Tumor Necrosis Factor-alpha; IL-1β, Interleukin-1 beta; IL-6, Interleukin-6; Neu, neutrophil; BM, bone marrow. The data are presented as mean ± SEM of three independent experiments. Individual data points [**(B, C, D, E, H, I)**] represent data from a single animal. Significance was calculated using unpaired two-tailed Student’s t-test **(B, C, D, E, H, I)**, cytokine measurements except IL-1β (BALF, lung tissue), Mann-Whitney U test for IL-1β in BALF and lung tissue and one-way ANOVA with Tukey’s multiple comparisons test **(K)**. *P < 0.05; **P < 0.01; ***P < 0.001; ****P < 0.0001.

To characterize the immune status following respiratory KP infection, mice were analyzed at 7 days post-infection, representing a post-acute phase after substantial bacterial clearance rather than peak active infection (**Figure 2A**). At this time point, bacterial loads in bronchoalveolar lavage fluid (BALF) and blood were nearly undetectable (**Figure 2B**). Nevertheless, TNF-α, IL-1β, and IL-6 remained elevated in bronchoalveolar lavage fluid (BALF) (**Figure 2C**), serum (**Figure 2D**), and lung tissue (**Figure 2E**), accompanied by persistent histopathological abnormalities (**Figure 2F**), indicating that inflammatory activity persisted despite minimal detectable bacterial burden.

Flow cytometric analysis (gating strategy shown in **Supplementary Figure 2**) revealed a statistically decrease in neutrophil frequency in bone marrow but the increase in the spleen was not statistically significant. (**Figures 2G–I**). These findings are consistent with a possible altered neutrophil distribution following respiratory infection, although absolute cell quantification would be required to distinguish redistribution from changes in neutrophil production.

**Figure 3:**
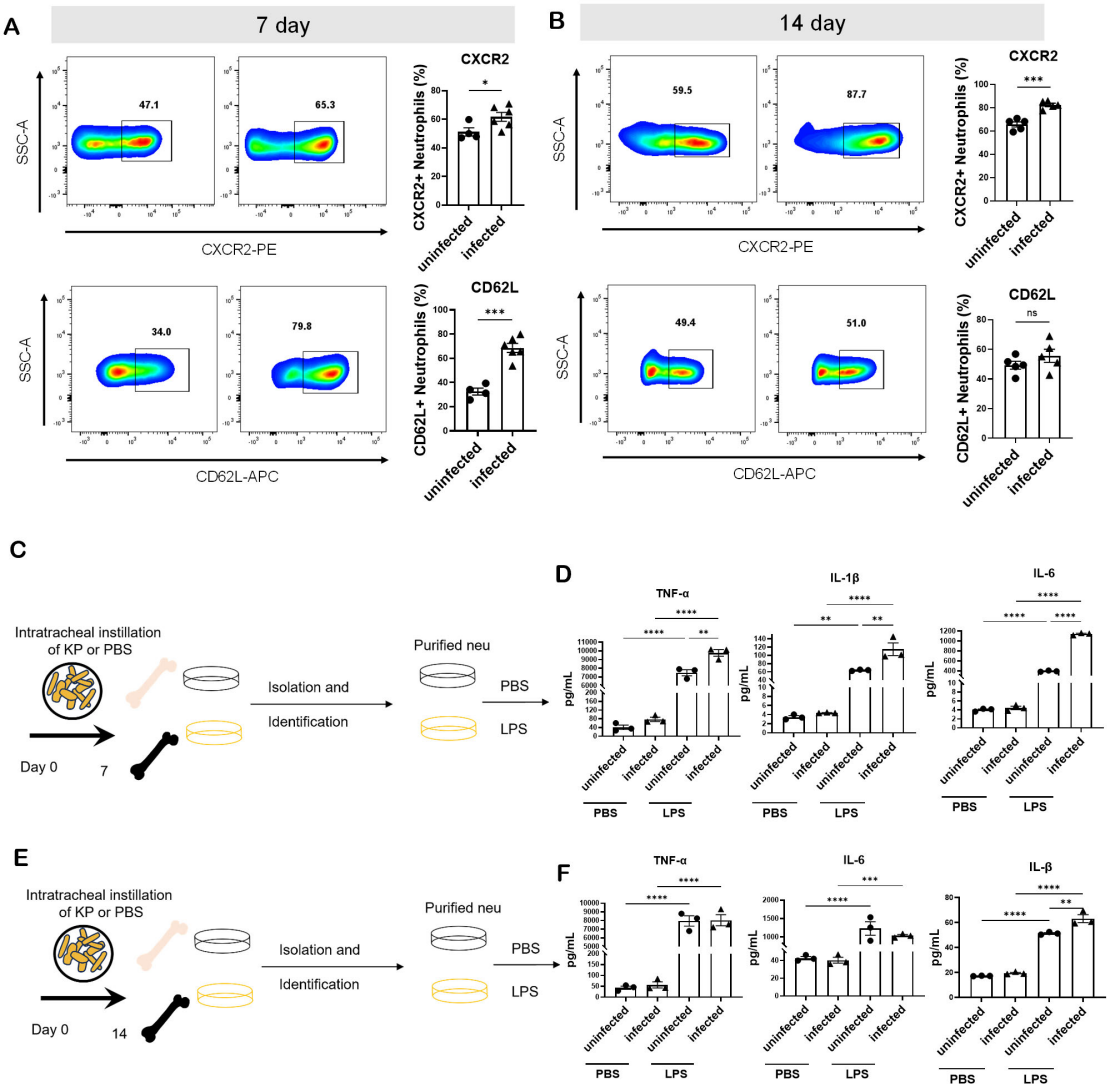
*Klebsiella pneumoniae* infection is associated with altered neutrophil phenotype and responsiveness. **(A, B)** Representative flow cytometric plots and quantification of CXCR2 and CD62L expression on bone marrow neutrophils isolated from mice at day 7 **(A)** or day 14 **(B)** after intratracheal instillation of Klebsiella pneumoniae (KP) (NTUH-K2044) or PBS. **(C–F)** Functional analysis of purified neutrophils after KP infection. Schematic diagrams showing the experimental design for neutrophil isolation at day 7 **(C)** and day 14 **(E)**. Purified neutrophils were stimulated ex vivo with PBS or LPS, and concentrations of TNF-α, IL-1β, and IL-6 in culture supernatants were measured at day 7 **(D)** and day 14 **(F)**. Data are presented as mean ± SEM. Each dot represents one mouse. Statistical analyses: unpaired two-tailed Student’s t-test for **(A, B)**; one-way ANOVA with Tukey’s multiple comparisons test for **(D, F)**. *P < 0.05, **P < 0.01, ***P < 0.001, ****P < 0.0001. KP, *Klebsiella pneumoniae*; i.t., intratracheal; PBS, phosphate-buffered saline; LPS, lipopolysaccharide.

We next asked whether this post-infectious inflammatory state was associated with altered neutrophil phenotype and responsiveness. At day 7 after infection, neutrophils exhibited increased proportions of CXCR2^+^ and CD62L^+^ subsets (**Figure 3A**). By day 14, increased CXCR2^+^ neutrophils remained detectable, suggesting that selected phenotypic changes persisted beyond the early post-infection phase (**Figure 3B**). To assess functional responsiveness, purified neutrophils were stimulated ex vivo with LPS (**Figures 3C, E**). Neutrophils from KP-infected mice at day 7 produced higher levels of TNF-α, IL-1β, and IL-6 than controls, whereas at day 14 only IL-1β remained elevated (**Figures 3D, F**).

To determine whether the enhanced allergic susceptibility persisted beyond the early post-infectious phase, PCA was induced 14 days after respiratory KP infection. Unlike the robust enhancement observed at day 7, KP-pretreated mice no longer exhibited significantly increased Evans blue extravasation or ear swelling compared with PCA-only controls at day 14 (**Supplementary Figure 4**). Together with the attenuation of neutrophil cytokine responses observed at this time point, these findings indicate that the enhanced allergic susceptibility is transient and coincides with the early post-infectious inflammatory state.

Collectively, these findings indicate that respiratory KP infection induces a transient post-infectious inflammatory state characterized by altered neutrophil phenotype and functional responsiveness. The attenuation of both neutrophil cytokine responses and enhanced PCA responses by day 14 further suggests that this immune modulation is time-dependent rather than persistently maintained.

### 3.3 Neutrophil depletion and adoptive transfer demonstrate a functional contribution of neutrophils to KP-enhanced PCA

**Figure 4:**
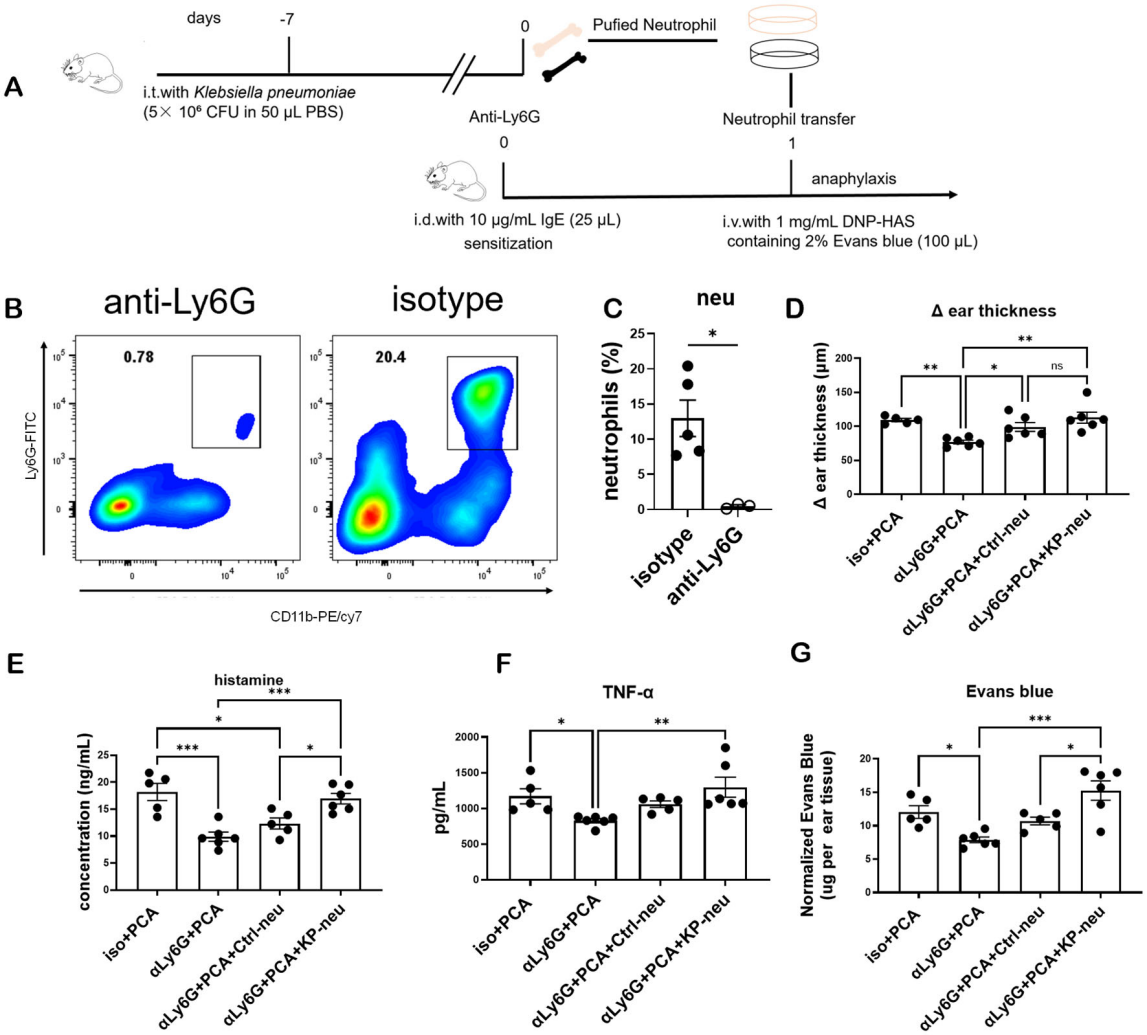
Neutrophil depletion and adoptive transfer validate the contribution of neutrophils to aggravated passive cutaneous anaphylaxis. **(A–C)** Schematic illustration of the neutrophil depletion and adoptive transfer strategy in the PCA model. Mice were intratracheally instilled with KP (NTUH-K2044) or PBS on day −7. For neutrophil depletion, mice received anti-Ly6G antibody before IgE sensitization. For adoptive transfer experiments, purified neutrophils were transferred before intravenous challenge with DNP-HSA containing 2% Evans blue. Representative flow cytometry plots **(B)** and quantification **(C)** showing the efficiency of neutrophil depletion after anti-Ly6G treatment. **(D–G)** Functional validation of neutrophil involvement in KP-enhanced PCA. Ear thickness **(D)**, histamine levels in ear tissue **(E)**, TNF-α levels in ear tissue **(F)**, and Evans blue extravasation normalized to ear weight **(G)** were measured in the indicated groups. Data are presented as mean ± SEM. Each dot represents one mouse. Statistical analyses: unpaired two-tailed Student’s t-test for **(C)**; one-way ANOVA with Tukey’s multiple comparisons test for **(D, E, G)**; Kruskal-Wallis test with Dunn’s multiple comparisons test for **(F)**. *P < 0.05, **P < 0.01, ***P < 0.001, ****P < 0.0001. KP, *Klebsiella pneumoniae*; PCA, passive cutaneous anaphylaxis; i.t., intratracheal; i.v, intravenous; PBS, phosphate-buffered saline; DNP-HSA, dinitrophenyl-human serum albumin. Iso, isotype control antibody; αLy6G, anti-Ly6G antibody-mediated neutrophil depletion; Ctrl-neu, neutrophils isolated from uninfected control donor mice; KP-neu, neutrophils isolated from KP-infected donor mice. All recipient mice were subjected to the PCA model. KP-neu indicates the donor source of transferred neutrophils and does not indicate KP pretreatment of recipient mice.

To determine whether neutrophils contribute functionally to KP-enhanced allergic responses, we performed depletion and adoptive transfer experiments (**Figure 4A**). Flow cytometric analysis confirmed that anti-Ly6G treatment substantially reduced circulating neutrophils at the analyzed time point (**Figures 4B, C**). Neutrophil depletion significantly attenuated PCA severity, as evidenced by reduced Evans blue extravasation, ear swelling, lowered TNF-α and histamine levels (**Figures 4D–G**), indicating that neutrophils are required for the full development of KP-enhanced allergic responses. Adoptive transfer of neutrophils restored these responses in neutrophil-depleted recipients, and neutrophils from KP-infected mice resulted in a greater enhancement across multiple PCA readouts than neutrophils from control donors (**Figures 4D–G**). These findings indicate that neutrophils exposed to respiratory KP infection acquire enhanced pro-inflammatory responsiveness that contributes to the amplification of subsequent allergic inflammation.

Together, these results indicate that neutrophils are functionally required for the enhanced PCA responses observed after KP infection. While these findings establish a functional contribution of neutrophils, they do not identify the downstream molecular pathways responsible for the amplification of allergic inflammation.

### 3.4 Prior KP infection differentially modulates cutaneous hypersensitivity responses across experimental models

**Figure 5:**
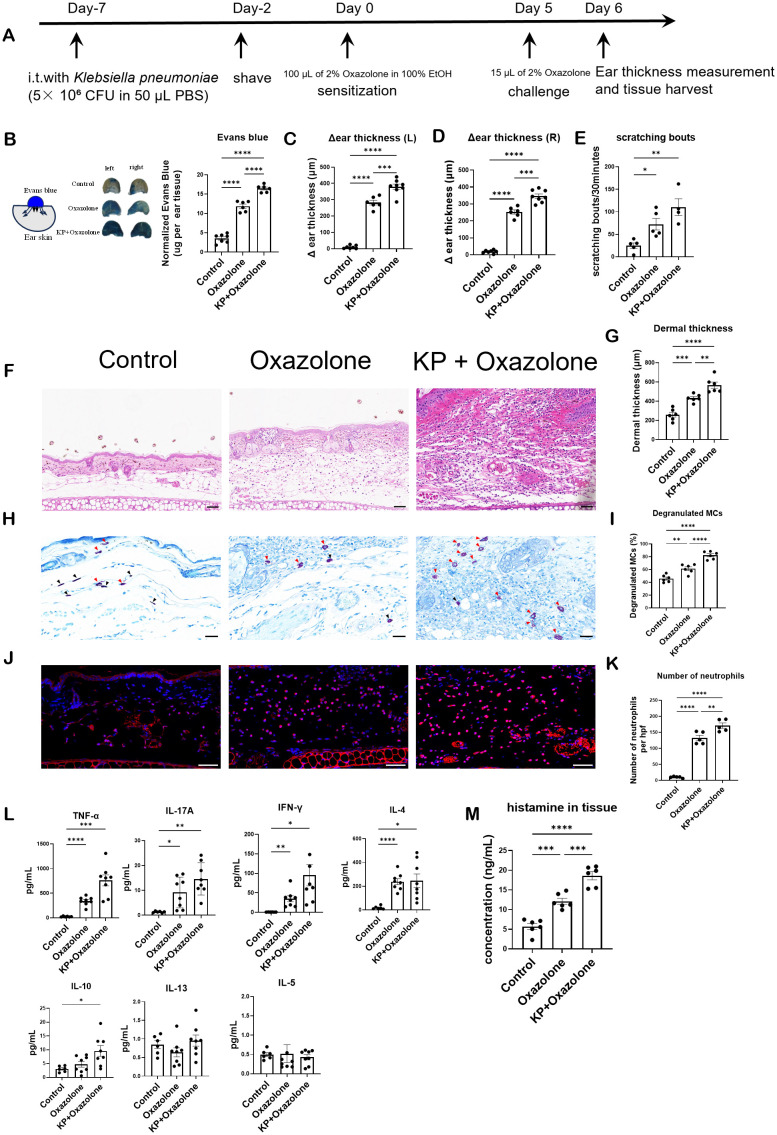
*Klebsiella pneumoniae* pretreatment exacerbates Oxazolone-induced skin inflammation in Mice. **(A)** Diagram showing the experimental design. **(B)** Representative ear images and the quantification of Evans blue dye (n = 6-7). **(C, D)** Ear thickness measurements after Oxazolone challenge (n = 7 - 8). **(E)** The number of scratching bouts observed over 30 min in mice 1 day post challenge, with the indicated hapten shown (n = 4 - 5). **(F, G)** Representative H&E-stained images **(F)**, and the quantification of dermal thickness measurements 24 hours after challenge **(G)** are shown (n = 6/group). Scale bars, 50 μm. **(H, I)** Representative Toluidine blue-stained images of MCs and the percentages of degranulated MCs in mouse ear (n = 6/group). Scale bars, 50 µm. **(J)** Immunofluorescent microscopic visualization with DAPI (blue) and Ly6G (magenta) to visualize neutrophils in ear skin. **(K)** Quantification of numbers of neutrophils is shown (n = 5/group). Scale bars, 50 µm. **(L)** Concentrations of key cytokines. (n = 7 - 8). **(M)** Concentration of histamine in mouse ear tissue (n = 6/group). Data are presented as mean ± SEM from three independent experiments, and each data point represents an individual animal. Control: solvents; Oxazolone: solvents with oxazolone sensitization and challenge; KP + Oxazolone: *Klebsiella pneumoniae* (NTUH-K2044) pretreatment followed by oxazolone sensitization and challenge. Significance was calculated using one-way ANOVA with Tukey’s multiple comparisons test [**(B, C, E, G, I, K, M)** and IL-13], by Welch one-way ANOVA with Dunnett’s T3 multiple comparisons test [**(D)**, TNF-α, IFN-γ, IL-17A, IL-4 and IL-10] or Kruskal-Wallis test with Dunn’s multiple comparisons test (IL-5). *P < 0.05; **P < 0.01; ***P < 0.001; ****P < 0.0001.

To determine whether this effect extends beyond IgE-mediated allergy, we examined oxazolone-induced contact hypersensitivity (**Figure 5A**). KP-pretreated mice exhibited greater Evans blue extravasation and ear swelling than oxazolone-only controls (**Figures 5B–D**). Increased scratching behavior was also observed as a supportive behavioral readout (**Figure 5E**); however, because this endpoint was assessed with a relatively small sample size, it should be interpreted cautiously and requires validation in larger cohorts. Histological analysis revealed more pronounced dermal thickening (**Figures 5F, G**), accompanied by elevated mast cell degranulation and neutrophil infiltration in lesional skin (**Figures 5H–K**). In addition, lesional tissues exhibited increased levels of TNF-α, IL-17A, IFN-γ, IL-10, IL-4, and histamine, whereas IL-5 and IL-13 were only limited altered (**Figures 5L, M**). These findings indicate that prior KP infection amplifies oxazolone-induced inflammation and is associated with a mixed inflammatory profile characterized by relatively stronger IL-17A-associated responses rather than a uniformly polarized Th2 response.

**Figure 6:**
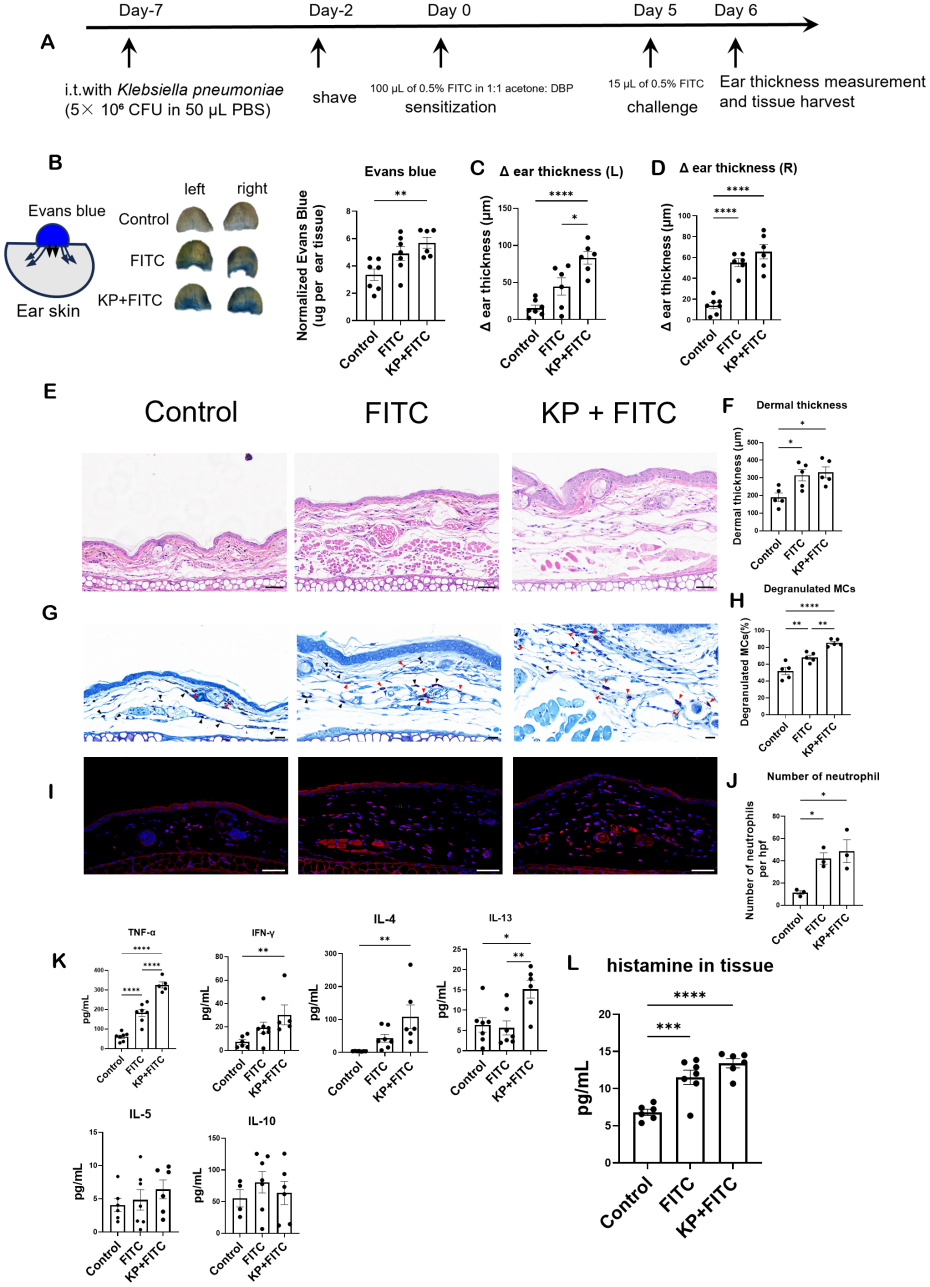
*Klebsiella pneumoniae* pretreatment selectively modulates FITC-induced skin inflammation in mice. **(A)** Schematic of the experimental timeline. Mice received an intratracheal (i.t.) administration of *Klebsiella pneumoniae* (NTUH-K2044) (5 × 10^6^ CFU in 50 µL PBS) on day -7. On day -2, the abdominal skin was shaved. Sensitization was performed on day 0 with 100 µL of 0.5% FITC dissolved in a 1:1 mixture of acetone and dibutyl phthalate (DBP) applied to the shaved abdominal skin. Challenge was delivered on day 5 with 15 µL of 0.5% FITC applied to both ears. On day 6, ear thickness was measured and tissues were harvested. **(B)** Representative photographs of mouse ears (left) and quantitative analysis of Evans blue extravasation (right) reflecting vascular leakage (n = 6 - 7). **(C, D)** Change in ear thickness (Δ thickness) were measured at indicated time points after challenge (n = 6 - 7). **(E)** Representative hematoxylin and eosin (H&E)-stained ear sections 24 hours after challenge, showing inflammatory infiltration. Scale bars, 50 µm. **(F)** Quantification of dermal thickness from sections in **(E)** (n = 5 per group). **(G)** Representative toluidine blue-stained images of mast cells (MCs) in ear skin. Scale bars, 50 µm. **(H)** Percentage of degranulated MCs (n = 5 per group). **(I)** Representative immunofluorescence images of neutrophils (Ly6G, magenta) and nuclei (DAPI, blue) in ear skin. Scale bars, 50 µm. **(J)** Quantification of neutrophil infiltration (n = 3 per group). **(K)** Concentrations of cytokines in ear tissue homogenates. **(L)** Histamine concentration in ear tissue (n = 6 - 7). Data are presented as mean ± SEM from three independent experiments, with each data point representing an individual animal. Experimental groups: Control (PBS), FITC (FITC, sensitization and challenge), and KP + FITC (KP pretreatment followed by FITC). Statistical analysis: one-way ANOVA with Tukey’s multiple comparisons test for **(C, D, E, H, J)**, TNF-α, IL-5, IL-4, IL-10, IL-13 and **(L)** and Kruskal-Wallis test with Dunn’s multiple comparisons test for **(B)** and IFN-γ. *P < 0.05; **P < 0.01; ***P < 0.001; ****P < 0.0001.

We next evaluated FITC-induced skin inflammation (**Figure 6A**). KP pretreatment modestly enhanced vascular permeability, ear swelling (**Figures 6B–D**), whereas dermal thickness was not significantly altered (**Figures 6E, F**). In parallel, mast cell degranulation was significantly increased, whereas neutrophil infiltration showed only a modest increase (**Figures 6G–J**). Because this quantification was based on a relatively small sample size, this endpoint should be interpreted as supportive rather than definitive evidence. Lesional tissues from KP + FITC mice displayed significantly higher levels of TNF-α and IL-13, while histamine was not significantly altered (**Figures 6K, L**), again suggesting that KP pretreatment modifies the inflammatory response without inducing a single dominant immune polarization.

Direct comparison of the three allergic models revealed that the impact of prior respiratory KP infection varied according to the inflammatory context. The most pronounced enhancement was observed in the PCA and oxazolone models, whereas FITC-induced inflammation exhibited a more limited response, affecting only selected inflammatory parameters. These findings indicate that respiratory KP infection does not uniformly amplify allergic inflammation but instead modulates distinct allergic responses in a model-dependent manner.

## 4 Discussion

By combining multiple models of cutaneous hypersensitivity, including IgE-mediated passive cutaneous anaphylaxis, oxazolone-induced contact dermatitis, and FITC-induced contact hypersensitivity, we show that prior respiratory *Klebsiella pneumoniae* infection can modulate distal allergic responses across distinct immunological contexts. This effect was observed after a substantial reduction in detectable bacterial burden, but at a stage when residual post-infectious inflammation was still detectable. Therefore, our findings are best interpreted as evidence of post-infectious immune modulation during the recovery phase, rather than definitive proof of stable long-term immune remodeling. Using neutrophil depletion and adoptive transfer approaches, we further identify neutrophils as important contributors to this process. Importantly, the magnitude and characteristics of this modulation varied according to the infecting bacterial strain, pathogen, timing after infection, and allergic model, indicating that the enhancement of distal allergic inflammation is context dependent rather than a universal consequence of respiratory bacterial infection.

The context-dependent nature of this response represents an important finding of the present study. Although prior KP infection broadly enhanced allergic responses in both the PCA and oxazolone models, the effects were more limited in FITC-induced hypersensitivity. Likewise, while the original NTUH-K2044 strain induced broad enhancement of allergic inflammation, the additional KP strains produced distinct response patterns, and *Pseudomonas aeruginosa* failed to reproduce the phenotype under the same experimental conditions. Together, these findings suggest that post-infectious immune modulation is shaped by both pathogen-specific characteristics and the local immunological context of the subsequent allergic response, rather than uniformly amplifying all forms of cutaneous inflammation.

The altered neutrophil distribution, surface phenotype, and cytokine responsiveness observed after KP infection may help explain the enhanced allergic responses in selected models. The reduced bone marrow neutrophil frequency and trend toward increased splenic neutrophils suggest post-infectious neutrophil mobilization or redistribution. Increased CXCR2^+^ neutrophils may indicate enhanced responsiveness to chemokine-driven recruitment, whereas altered CD62L expression may reflect changes in neutrophil trafficking or activation status. In parallel, the increased production of TNF-α, IL-1β, and IL-6 after ex vivo stimulation suggests that neutrophils remain in a heightened inflammatory state during the post-infection phase. Consistent with this interpretation, both neutrophil cytokine responsiveness and enhanced PCA responses were attenuated by day 14 after infection, supporting the concept that these changes represent transient post-infectious immune modulation rather than persistently maintained immune remodeling.

Although peripheral blood flow cytometry confirmed effective depletion of circulating neutrophils after anti-Ly6G treatment, depletion efficiency was not systematically assessed in other tissue compartments, such as spleen or inflamed ear tissue. Therefore, incomplete depletion or tissue-specific differences in depletion efficiency cannot be fully excluded. Potential antibody-mediated indirect effects should also be considered when interpreting the depletion experiment. Nevertheless, the attenuation of KP-enhanced PCA responses after anti-Ly6G treatment, together with the restoration of these responses after neutrophil adoptive transfer, supports an important functional contribution of neutrophils in this experimental setting, although these experiments do not define the downstream molecular mechanisms responsible or allergic amplification.

Our results also provide insight into the role of innate immune cells in post-infectious immune modulation. Neutrophils from previously infected mice exhibited enhanced production of pro-inflammatory cytokines upon secondary stimulation even after a substantial reduction in detectable bacterial burden, consistent with altered functional responsiveness during the post-infectious inflammatory phase. Although these findings are compatible with infection-associated neutrophil functional priming, they do not constitute direct evidence of stable neutrophil reprogramming or trained immunity. Given the short lifespan of circulating neutrophils, the persistence of these functional alterations may involve changes in granulopoiesis or the inflammatory bone marrow environment (38–40). However, the upstream mechanisms responsible for these observations remain to be determined.

Notably, the impact of prior infection on subsequent allergic responses varies across models. In both the IgE-driven PCA model and the oxazolone-induced dermatitis model with mixed inflammatory features, KP pretreatment resulted in a broad amplification of inflammatory responses. In contrast, in the FITC-induced contact hypersensitivity model, we observed a partial dissociation between microscopic inflammatory changes and macroscopic tissue swelling. These findings suggest that prior KP infection modulates allergic inflammation in a context-dependent manner, likely influenced by the local immune environment of each model, rather than uniformly amplifying all allergic responses.

In our study, KP-pretreated mice showed concurrent increases in TNF-α, mast cell degranulation, histamine, and neutrophil infiltration in allergic skin lesions. Rather than implying a direct causal relationship between these observations, our findings suggest that these events occur in parallel within a broader inflammatory milieu established after respiratory infection. This interpretation is consistent with recent studies demonstrating concurrent neutrophil recruitment, mast cell activation, and TNF expression during cutaneous inflammation (36), while the precise interactions among these components remain to be elucidated. Accordingly, the proposed neutrophil-mast cell interaction should be regarded as a mechanistic hypothesis requiring direct experimental testing.

Several limitations should be acknowledged. 1) This study cannot fully distinguish transient post-infectious immune modulation from persistent immune remodeling. Although allergic challenges were initiated after a substantial reduction in detectable bacterial burden, residual cytokine elevation and lung histopathological changes remained detectable at day 7. Moreover, residual bacterial products may still contribute to the inflammatory milieu despite minimal detectable bacterial burden. Although selected neutrophil phenotypes and PCA responses were additionally examined at day 14 and showed substantial attenuation, longer-term analysis will be required to define the duration and reversibility of these immune changes. Future transcriptomic, epigenetic, metabolic, and hematopoietic studies will be also necessary to define their underlying mechanisms. 2) Although neutrophil depletion and adoptive transfer support a functional contribution of neutrophils, these experiments do not identify the specific neutrophil-derived mediators or cellular interactions responsible for allergic amplification. We also acknowledge that CXCL1, CXCL2, and CXCL5—key chemokines for neutrophil recruitment—were not examined in this study. Future studies incorporating chemokine profiling or functional blockade will help define their contribution to this model. Moreover, transferred neutrophils were not directly labeled or tracked *in vivo*, so their persistence, trafficking, and localization remain undefined. 3) Although we expanded our analysis by including additional *K. pneumoniae* strains and a comparison with *Pseudomonas aeruginosa*, the generalizability of these findings should still be interpreted cautiously. Additional respiratory pathogens, both sexes, longer post-infection intervals, and broader allergic disease models should be examined to define the scope and underlying mechanisms of post-infectious allergic modulation.

## 5 Conclusion

Collectively, our findings suggest that prior respiratory *Klebsiella pneumoniae* infection induces a transient post-infectious immune state associated with altered neutrophil responsiveness and enhance distal allergic inflammation. This phenomenon is pathogen-, strain-, time-, and model-dependent, with the strongest effects observed in IgE-mediated PCA and oxazolone-induced inflammation, but more limited effects in FITC-induced hypersensitivity. Together, these findings support a model in which respiratory KP infection transiently modifies systemic immune responsiveness and thereby shapes subsequent allergic inflammation in a context-dependent manner.

## Data Availability

The original contributions presented in the study are included in the article/**Supplementary Material**. Further inquiries can be directed to the corresponding author.
