# Supplementary material for: Respiratory Klebsiella pneumoniae infection transiently alters neutrophil responsiveness and enhances distal allergic inflammation
Source: Front Immunol. 2026 Aug 4;17:1853024. doi: 10.3389/fimmu.2026.1853024 (PMC13481219; doi:10.3389/fimmu.2026.1853024)
Supplement: Supplementary file 1 [file DataSheet1.pdf]

frontiers

The International Society for the Advancement of Cytometry (ISAC) has highlighted the importance of including comprehensive methodological information to ensure data reproducibility and reliability. In line with this, Frontiers in Immunology now requires authors to submit a checklist for manuscripts that involve flow or mass cytometry. This checklist helps standardize the reporting process, improving the quality and transparency of published data. By doing so, we support scientific progress, making it easier for other researchers to replicate and validate experiments.

This form should be submitted with any manuscripts using flow or mass cytometry.

Sample/specimen/material description

- [ ] Total blood
- [ ] PBMCs
- [x] Organ digests

Other Bone marrow flush and spleen

Did the samples suffer any treatment before or after incubation with the antibodies?

- [ ] Drug
- [ ] Cell permeabilization
- [x] Dye Samples were stained with Zombie Aqua Fixable Viability Kit (BioLegend) before surface antibody staining.
- [ ] Propidium iodine
- [ ] Not applicable

Other

Instrument and antibodies

Name of the Cytometer Navios (Beckman Coulter)

Antibodies and targets	Fluorochrome/ Metal	Catalog number/Company
e.g. anti-CD4	FITC	Cat. XXX/ XXX ltd.
CD45	PerCP	103129 / BioLegend

CD11b	PE/Cy7	101215 / BioLegend
Ly6G	FITC	127605 / BioLegend
CXCR2	PE	149303 / BioLegend
CD62L	APC	104411 / BioLegend
Zombie Aqua		423101 / BioLegend
anti-mouse CD16/32		101319/ BioLegend

Data analyses

1. Name of the software FlowJo (Tree Star Inc.)
2. Reference gating strategy in the manuscript or supplementary material

Gating strategy in (eg Figure X) Gating strategy is shown in Figure S2
