# Supplementary material for: Respiratory Klebsiella pneumoniae infection transiently alters neutrophil responsiveness and enhances distal allergic inflammation
Source: Front Immunol. 2026 Aug 4;17:1853024. doi: 10.3389/fimmu.2026.1853024 (PMC13481219; doi:10.3389/fimmu.2026.1853024)
Supplement: Supplementary file 2 [file DataSheet2.docx]

**Supplementary Material**

**Supplementary Figure 1. Representative flow cytometry plots for purity verification of bone marrow neutrophils.**

Cells were first gated based on FSC/SSC profiles to exclude debris, followed by singlet selection. Neutrophils were then identified as CD11b⁺Ly6G⁺ cells, which accounted for approximately 90% of the analyzed single-cell population, confirming the purity of the isolated neutrophil preparations.

Left: naïve mouse; right: *Klebsiella pneumoniae*‑infected mouse. Neutrophils were gated as CD11b⁺Ly6G⁺ cells. Purity exceeded 90% in both groups.

**Supplementary Figure 2. Gating strategy for flow cytometric identification of neutrophils in bone marrow and spleen.**

Single-cell suspensions from bone marrow (A) and spleen (B) were analyzed by flow cytometry. The sequential gating strategy was as follows: total cells were first gated on forward scatter area (FSC‑A) versus side scatter area (SSC‑A) to exclude debris; single cells were then selected using FSC‑height (FSC‑H) versus FSC‑A to discriminate doublets; viable cells were gated as Zombie Aqua‑negative (Zombie Aqua^-^); leukocytes were identified as CD45^+^ cells; finally, neutrophils were defined as CD45^+^CD11b^+^Ly6G^+^. Numbers indicate the percentage of cells at each gating step.

**Supplementary Figure 3.** **Respiratory bacterial pretreatment modulates IgE-elicited passive cutaneous anaphylaxis in a pathogen- and strain-dependent manner.**

(A) Schematic diagram of the experimental design. Mice were intratracheally treated with PBS, Pseudomonas aeruginosa PAO1, KP1, or KP2 on day 0, followed by PCA induction on day 7 and anaphylaxis challenge on day 8. (B) Representative ear images and the quantification of Evans blue dye (n = 6 - 8/group). (C and D) Changes in ear thickness of the left ear (C) and right ear (D) are shown. Data are presented as mean ± SEM. Control: PBS; PCA: PBS + IgE/DNP-HSA; PAO1 + PCA: *Pseudomonas aeruginosa* PAO1 + IgE/DNP-HSA; KP1 + PCA: *Klebsiella pneumoniae* ATCC BAA1705/ST258 + IgE/DNP-HSA; KP2 + PCA: *Klebsiella pneumoniae* ATCC BAA2146/ST11 + IgE/DNP-HSA. PCA, passive cutaneous anaphylaxis; i.t., intratracheal; i.d., intradermal; i.v., intravenous; PAO1, *Pseudomonas aeruginosa* PAO1 ATCC 15692; KP1, *Klebsiella pneumoniae* ATCC BAA1705/ST258; KP2, *Klebsiella pneumoniae* ATCC BAA2146/ST11. Individual data points represent data from a single animal. Significance was calculated using one-way ANOVA with Tukey’s multiple comparisons test. **P* < 0.05; ***P* < 0.01; ****P* < 0.001; *****P* < 0.0001.

**Supplementary Figure 4. The PCA-enhancing effect of respiratory KP pretreatment is not sustained at a later post-infection time point.**

(A) Schematic diagram of the experimental design. Mice were intratracheally treated with PBS or *Klebsiella pneumoniae* on day 0. PCA was induced on day 14 by intradermal injection of anti-DNP IgE, followed by intravenous challenge with DNP-HSA containing Evans blue on day 15. (B) Quantification of Evans blue dye extravasation in ear tissue. (C and D) Changes in ear thickness of the left ear (C) and right ear (D) are shown. Data are presented as mean ± SEM. Control: PBS; PCA: PBS + IgE/DNP-HSA; KP + PCA: KP + IgE/DNP-HSA. PCA, passive cutaneous anaphylaxis; i.t., intratracheal; i.d., intradermal; i.v., intravenous; KP (NTUH-K2044), *Klebsiella pneumoniae*. Individual data points represent data from a single animal. Significance was calculated using one-way ANOVA with Tukey’s multiple comparisons test. ns, not significant; **P* < 0.05; ***P* < 0.01; ****P* < 0.001; *****P* < 0.0001.

**Characterization of bacterial strain backgrounds**

To further define the bacterial backgrounds used in the validation experiments, we performed additional strain characterization. *K. pneumoniae* ATCC BAA-1705 (ST258), *K. pneumoniae* ATCC BAA-2146 (ST11), and *Pseudomonas aeruginosa* PAO1(ATCC15692) were kindly provided by the Microbiology Laboratory of the Center for Clinical Laboratories, Affiliated Hospital of Guizhou Medical University. For *K. pneumoniae*, we examined the hypermucoviscosity phenotype of the three strains using the string test and, by PCR, screened for nine virulence-associated genes (*iroB*, *peg-344*, *rmpA*, *rmpA2*, *mrkD*, *entB*, *ybtS*, *fimH*, and *wcaG*) and two carbapenemase resistance genes (*blaKPC* and *blaNDM*). For *P. aeruginosa* PAO1, we examined four type III secretion system-associated virulence genes (*exoS*, *exoT*, *exoU,* and *exoY*).

PCR analysis showed that NTUH-K2044 carried classical hypervirulence-associated genes, including *rmpA*, *rmpA2*, *peg-344*, *iroB*, *wcaG*, and *ybtS*. In contrast, BAA-1705 and BAA-2146 lacked these hypervirulence-associated genes but carried distinct carbapenemase resistance genes: *blaKPC* in BAA-1705 and *blaNDM* in BAA-2146. Common colonization-associated genes, including *mrkD*, *entB*, and *fimH*, were detected in all three *K. pneumoniae* strains. The string test was positive only for the hypervirulent NTUH-K2044 strain, whereas BAA-1705 and BAA-2146 were negative (Table. 1 and Supplementary Figure 5). As a non-Klebsiella control, *P. aeruginosa* PAO1 showed the expected type III secretion system genotype, *exoS* ⁺/*exoT* ⁺/*exoY* ⁺/*exoU* ⁻, consistent with its reported model-strain characteristics (Table.2 and Supplementary Figure 6). Together, these data confirmed that the validation experiments included bacteria with distinct species, virulence, and resistance backgrounds.

**Table. 1** PCR detection of virulence and resistance genes in *Klebsiella pneumoniae* strains, and string test results

| Gene | K. pneumoniae | | | Primer Sequence | Amplified fragment  (bp) |
| --- | --- | --- | --- | --- | --- |
|  | BAA1705 | BAA2146 | NTUH-K2044 |  |  |
| rmpA | **-** | **-** | **+** | F:CATAAGAGTATTGGTTGACAG | 461 |
|  |  |  |  | R:CTTGCATGAGCCATCTTTCA |  |
| rmpA2 | **-** | **-** | **+** | F:CTTTATGTGCAATAAGGATGT | 430 |
|  |  |  |  | R: CCTCCTGGAGAGTAAGCATT |  |
| peg-344 | **-** | **-** | **+** | F:CTTGAAACTATCCCTCCAGTC | 508 |
|  |  |  |  | R:CCAGCGAAAGAATAACCCC |  |
| *IroB* | **-** | **-** | **+** | F: ATCTCATCATCTACCCTCCGCTC | 235 |
|  |  |  |  | R: GGTTCGCCGTCGTTTTCAA |  |
| wcaG | **-** | **-** | **+** | F:GGTTGGKTTCAGCAATCGTA | 169 |
|  |  |  |  | R:ACTATTCCGCCAACTTTTGC |  |
| mrkD | **+** | **+** | **+** | F:AAGCTATCGTACTTCCGGCA | 340 |
|  |  |  |  | R:GGCGTTGGCGCTCAGATAGG |  |
| entB | **+** | **+** | **+** | F:GTCAACTGGGCCTTTGAGCCGTC | 400 |
|  |  |  |  | R:TATGGGCGTAAACGCCGGTGAT |  |
| ybtS | **-** | **-** | **+** | F:GACGGAAACAGCACGGTAAA | 242 |
|  |  |  |  | R:GAGCATAATAAGGCGAAAGA |  |
| fimH | **+** | **+** | **+** | F:TGCTGCTGGGCTGGTCGATG | 506 |
|  |  |  |  | R:GGGAGGGTGACGGTGACATC |  |
| bla*KPC* | **+** | **-** | **-** | F:GCTACACCTAGCTCCACCTTC | 989 |
|  |  |  |  | R:ACAGTGGTTGGTAATCCATGC |  |
| *blaNDM* | **-** | **+** | **-** | F:GGTTTGGCGATCTGGTTTTC | 621 |
|  |  |  |  | R:CGGAATGGCTCATCACGATC |  |
| String length (mm) | Negative | Negative | Positive | >5 mm | |

**Supplementary Figure 5.** Gel electrophoresis of PCR products for virulence and resistance genes in three *Klebsiella pneumoniae* strains. ① ATCCBAA1705, ②ATCCBAA2146, ③NTUH-K2044.

**Table. 2** PCR detection of virulence genes in *Pseudomonas aeruginosa* PAO1

| Gene | Result | Primer Sequence | Amplified fragment (bp) |
| --- | --- | --- | --- |
| *exoS* | **+** | F:CGTCGTGTTCAAGCAGATGGTGCTG | 444 |
|  |  | R:CCGAACCGCTTCACCAGGC |  |
| *exoT* | **+** | F:CAATCATCTCAGCAGAACCC | 1159 |
|  |  | R:TGTCGTAGAGGATCTCCTG |  |
| *exoU* | **-** | F:ATG CAT ATC CAA TCG TTG | 2000 |
|  |  | R:TCA TGT GAA CTC CTT ATT |  |
| *exoY* | **+** | F:TATCGACGGTCATCGTCAGGT | 1035 |
|  |  | R:TTGATGCACTCGACCAGCAAG |  |

**Supplementary Figure 6**. Gel electrophoresis of PCR products for virulence in *Pseudomonas aeruginosa* PAO1.

**Supplementary Methods**

1. Virulence and Resistance Gene Identification

Virulence and resistance genes were identified by PCR. For *K. pneumoniae*, nine virulence genes (*iroB,* *peg-344*, *rmpA*, *rmpA2*, *mrkD*, *entB*, *ybtS*, *fimH*, and *wcaG*) and two carbapenemase genes (*blaKPC* and *blaNDM*) were targeted. For *P. aeruginosa* PAO1, the type III secretion system (T3SS) effector genes *exoS*, *exoT*, *exoY*, and *exoU* were detected. PCR products were analyzed by Gel electrophoresis to identify target bands.

2. String Test

A single bacterial colony was picked from an agar plate using an inoculation loop and lifted vertically. A positive string test was defined as a string >5 mm in length, indicating a hypermucoviscous phenotype of *Klebsiella pneumoniae*.
